# A qualitative study on healthcare providers’ biases towards transgender and gender diverse people accessing contraceptive care

**DOI:** 10.1080/26410397.2025.2577002

**Published:** 2025-10-24

**Authors:** Yasaman Zia, Connie Folse, Adrien Lawyer, Felix Zeid, Alejandra Alvarez, Erica Somerson, Kathryn Albergate Davis, Dane Menkin, Mitzi Hawkins, Jen Hastings, Cynthia Harper

**Affiliations:** a Assistant Professional Researcher, Department of Obstetrics, Gynecology, and Reproductive Sciences, University of California San Francisco, San Francisco, CA, USA; Researcher, Bixby Center for Global Reproductive Health,University of California San Francisco, San Francisco, CA, USA.; b Health Educator, Department of Obstetrics, Gynecology, and Reproductive Sciences, University of California San Francisco, San Francisco, CA, USA; Senior Manager Curriculum & Training, Bixby Center for Global Reproductive Health, University of California San Francisco, San Francisco, CA, USA; c Co-Director, Transgender Resource Center of New Mexico, Albuquerque, NM, USA; d Lead Health Education Coordinator, UNM Medical Group, University of New Mexico (UNM) Truman Health Services, Albuquerque, NM, USA; e Data Manager/Analyst, Department of Obstetrics, Gynecology, and Reproductive Sciences, University of California San Francisco, San Francisco, CA, USA; Data Manager/Analyst, Bixby Center for Global Reproductive Health, University of California San Francisco, San Francisco, CA, USA; f Health Educator, Department of Obstetrics, Gynecology, and Reproductive Sciences, University of California San Francisco, San Francisco, CA, USA; Training & Technical Assistance Manager, Bixby Center for Global Reproductive Health, University of California San Francisco, San Francisco, CA, USA; g Consultant, Department of Obstetrics, Gynecology, and Reproductive Sciences, University of California San Francisco, San Francisco, CA, USA; Contraceptive Training Specialist, Bixby Center for Global Reproductive Health, University of California San Francisco, San Francisco, CA, USA; h Family Nurse Practitioner, Director of LGBTQ Services, Mainline Health, Philadelphia, PA, USA; i Assistant Professor, Department of Obstetrics, Gynecology, and Reproductive Sciences, University of California San Francisco, San Francisco, CA, USA; j Assistant Clinical Professor, Department of Family and Community Medicine, University of California San Francisco, San Francisco, CA, USA; k Professor, Department of Obstetrics, Gynecology, and Reproductive Sciences, University of California San Francisco, San Francisco, CA, USA; Investigator, Bixby Center for Global Reproductive Health, University of California San Francisco, San Francisco, CA, USA

**Keywords:** transgender health, gender-affirming, provider bias, contraception, gender diverse

## Abstract

Bias in sexual and reproductive health care (SRH) undermines the quality and delivery of essential services. For transgender and gender diverse (TGD) patients, barriers to care may be acute when seeking gendered services, such as contraception. Few studies have assessed providers’ perceptions of TGD patients in SRH. Our objective was to examine bias in contraceptive providers’ attitudes towards and experiences with TGD patients. We conducted in-depth interviews, from August 2022 to August 2024, with 41 healthcare providers located throughout the US and attending CME-accredited trainings on contraceptive counselling. We utilised deductive thematic analysis to identify the domains of implicit and explicit bias specific to TGD patients. Many providers struggled to understand gender diversity and had difficulty using gender-inclusive frameworks in care delivery. Explicit biases were exemplified in the pervasiveness of gender binarism within the healthcare system and providers’ lack of experience with TGD patients. Providers demonstrated implicit biases through their deficits in knowledge regarding TGD patients’ medical needs and culturally insensitive approaches. They offered suggestions to mitigate bias, including institutional changes to make clinics more trans-inclusive and affirming. Biases and disparities specific to TGD patients are perpetuated through a lack of awareness and stigmatisation in healthcare settings. Our findings highlight areas to improve awareness of bias, dispel confusion with evidence on gender-inclusive care, and integrate structural changes within clinics to reduce the burdensome impacts of bias on TGD patients. Advocacy at both the provider and institutional levels is needed to offer quality contraceptive care for TGD patients.

## Plain language summary

Bias and discrimination in sexual and reproductive health care reduce the quality and accessibility of essential services. Transgender and gender diverse (TGD) patients face many barriers and biases when seeking contraceptive care. We conducted interviews with healthcare providers working in contraceptive care to assess different types of bias in their experiences with TGD patients. We found that many providers struggled to understand gender identity and to use gender-inclusive frameworks in care delivery. Biases were present within the healthcare system itself and further perpetuated through providers’ lack of experience with TGD patients’ medical needs, pronouns and gender identities. They also brought up solutions to mitigate bias, including institutional changes to make clinics more trans-inclusive and affirming. Our findings highlight areas to improve awareness of bias, reduce confusion with evidence on gender-inclusive care and incorporate structural changes within clinics to reduce the burdensome impacts of bias on transgender and gender diverse patients.

## Résumé

Les préjugés en matière de santé sexuelle et reproductive (SSR) compromettent la qualité et la prestation des services essentiels. Pour les patients transgenres et de genre divers, les obstacles à l’accès aux soins peuvent être importants lorsqu’ils recherchent des services genrés, comme la contraception. Peu d’études ont évalué le point de vue des prestataires de soins sur les patients transgenres et de genre divers en SSR. Notre objectif était d’examiner les préjugés dans les attitudes et les expériences des prestataires de contraception avec ces patients. Nous avons mené des entretiens approfondis, d’août 2022 à août 2024, avec 41 prestataires de soins de santé répartis sur tout le territoire des États-Unis d’Amérique et qui participaient à des formations agréées par la formation médicale continue (FMC) sur le conseil en contraception. Nous avons utilisé l’analyse thématique déductive pour identifier les domaines des préjugés implicites et explicites propres aux patients transgenre et de genre divers. Beaucoup de prestataires avaient du mal à comprendre la diversité de genre et à utiliser des cadres inclusifs en matière de genre dans la prestation des soins. Les préjugés explicites étaient illustrés par la généralisation du binarisme de genre au sein du système de santé et le manque d’expérience des prestataires avec les patients transgenre et de genre divers. Les prestataires ont fait preuve de préjugés implicites par leur manque de connaissances concernant les besoins médicaux des patients transgenre et de genre divers et leurs approches culturellement insensibles. Ils ont proposé des solutions pour atténuer ces préjugés, notamment des changements institutionnels visant à rendre les centres de santé plus inclusifs et plus valorisants. Les préjugés et les disparités propres aux patients transgenres et de genre divers sont perpétués par le manque de sensibilisation et la stigmatisation dans les établissements de santé. Nos résultats mettent en évidence les domaines dans lesquels il convient de mieux prendre conscience des préjugés, de dissiper la confusion avec des données sur les soins inclusifs en matière de genre et d’intégrer des changements structurels au sein des centres pour réduire les conséquences néfastes des préjugés sur les patients transgenres et de genre divers. Un plaidoyer, tant au niveau des prestataires qu’au niveau institutionnel, est nécessaire pour offrir des soins contraceptifs de qualité aux patients transgenres et de genre divers.

## Resumen

Los prejuicios en los servicios de salud sexual y reproductiva (SSR) socavan la calidad y la prestación de servicios esenciales. Las pacientes transgénero y de género diverso (TGD) podrían enfrentar barreras agudas durante su búsqueda de servicios sensibles al género, tales como los servicios de anticoncepción. Pocos estudios han evaluado las percepciones de los prestadores de servicios sobre pacientes TGD en SSR. Nuestro objetivo era examinar los prejuicios en las actitudes de prestadores de servicios de anticoncepción hacia pacientes TGD y sus experiencias con esas pacientes. Entre agosto de 2022 y agosto de 2024, realizamos entrevistas a profundidad con 41 prestadores de servicios de salud ubicados en diferentes partes de Estados Unidos que asistieron a capacitaciones sobre consejería anticonceptiva acreditadas por el consejo de acreditación de educación médica continua. Utilizamos análisis temático deductivo para identificar los dominios de prejuicios implícitos y explícitos relacionados específicamente con pacientes TGD. A muchos prestadores de servicios les resultó difícil entender la diversidad de género y utilizar marcos inclusivos de género en la prestación de servicios. Ejemplos de prejuicios explícitos son la omnipresencia del binarismo de género en el sistema de salud y la falta de experiencia de los prestadores de servicios con pacientes TGD. Los prestadores de servicios demostraron prejuicios implícitos en sus déficits de conocimiento sobre las necesidades médicas de pacientes TGD y en sus enfoques insensibles culturalmente. Ofrecieron sugerencias para mitigar los prejuicios, tales como cambios institucionales para lograr que los centros de salud sean más inclusivos y afirmadores de personas trans. Los prejuicios y las disparidades relacionados específicamente con pacientes TGD son perpetuados mediante la falta de conocimiento y la estigmatización en ámbitos de salud. Nuestros hallazgos destacan las áreas para mejorar la conciencia de prejuicios, disipar la confusión con evidencia sobre la atención inclusiva de género e integrar cambios estructurales en los centros de salud para reducir los impactos gravosos de los prejuicios en pacientes TGD. Se necesita incidencia política tanto a nivel de los prestadores de servicios como a nivel institucional para ofrecer servicios de anticoncepción de calidad para pacientes TGD.

## Background

Bias in sexual and reproductive health care undermines the quality and person-centred delivery of essential services. Transgender and gender diverse (TGD)^1^ communities face multilevel barriers when accessing healthcare services.^2^ Both qualitative and quantitative studies have assessed the hostility that TGD patients encounter through interactions with staff, nurses, medical providers, as well as healthcare systems that perpetuate discrimination and stigma.^3–6^ Discrimination can be expressed through either implicit biases, or the unconscious mental processes that evaluate others unfavourably and have a significant impact on decision-making, or explicit biases, or the conscious preferences, beliefs and attitudes that one expresses.^7–9^ Both international and regional human rights treaty bodies affirm the right to health care for TGD people.^10^ However, the sociopolitical hostility towards TGD people both globally and in the US reinforces discrimination and oppression through wide-ranging laws that deny their human rights and also medical care in restricting access to gender-affirming care.^11^ Additionally, the current international treaties outlining reproductive rights and access to contraception focus solely on cisgender women of reproductive age and systemically ignore TGD patients.^12^ Contraceptive care spaces are a critical avenue for upholding TGD patients’ rights to access sexual and reproductive health care.

In a model of minority stress specific to sexual and gender minorities, the combination of stress with the absence of social safety co-creates cumulative inequalities that perpetuate mental health and physical health disparities.^13^ TGD patients experience bias in healthcare systems in the form of cissexism, gender binarism and heteronormative patterns through which providers may refuse care, misgender or harm TGD patients^2,3,6^ (see Box 1 for glossary).

**Box 1. Glossary of terms**

**Cisnormative** – belief that the most normal gender identity is cisgender, meaning that a person's lived gender matches the sex they were assigned at birth based on physical sex characteristics^14^

**Cissexism** – bias or prejudice that favours cisgender people^15^

**Gender affirmation** – refers to being recognised or affirmed in a person's gender identity. It is usually conceptualized as having social, psychological, medical, and legal dimensions.^1^

**Gender binarism** – refers to the idea there are two and only two genders, men and women; the expectation that everyone must be one or the other; and that all men are males, and all women are females.^1^

**Heteronormative** – the belief that being heterosexual is the most desirable and normal sexual identity^14^

These patterns are parallelled at the institutional level to enact barriers and “erase” TGD patients’ identities and experiences in health care.^3,16^ In one study utilising an implicit association test, healthcare providers exhibited higher levels of bias than the general population.^4^ In the 2022 US Transgender Survey of nearly 100,000 TGD people, almost half (48%) report mistreatment from their healthcare providers within the past year, and 24% indicate that they had either delayed or avoided seeking care for fear of mistreatment and discrimination.^17^ Despite similar Ob/Gyn guidelines applying to these patients, one study found that while 80% of cisgender women had visited an Ob/Gyn in the past year, that rate was only 40% for transmen.^18^ Together, these effects delay and prevent TGD patients from seeking needed healthcare services,^19–21^ and delayed health care can contribute to negative health outcomes, exacerbate health disparities and have far-reaching public health implications.

Gender affirmation is defined by the World Professional Association for Transgender Health as “a process of recognising or affirming TGD people in their gender identity – whether socially, medically, legally, behaviourally, or some combination of these”.^1^ Gender-affirming care entails, but is not limited to, hormonal treatments to assist with transition, hormonal contraception to suppress menstruation and reproductive technologies, as well as surgeries.^1,22–24^ Delivering gender-affirming care also requires collaborative patient–provider decision-making alongside the use of language and terminology that is culturally responsive, including gender-aligned pronouns, the names TGD patients choose and the use of patient-led language to refer to body parts.^1,25^

TGD people have been largely excluded from clinical care and research, contributing to providers’ lack of knowledge of gender-affirming approaches to sexual and reproductive care.^16,26^ TGD people are reproductive agents who may desire fertility and/or contraception.^22,25–28^ For those pursuing contraception, barriers to care may be acute when seeking gendered services – navigating contraceptive guidelines, recommendations, and services that are frequently oriented towards heterosexual cisgender women may be challenging.^22,28,30,31^ In addition, healthcare providers frequently misunderstand TGD patients’ capacity for pregnancy and contraceptive eligibility.^22,26–28^ Few studies have assessed the manifestations of anti-transgender bias among providers in contraceptive and reproductive health care.^5,32–34^ Our objective was to examine anti-transgender bias in contraceptive care providers’ perceptions of, and experiences with, TGD patients.

## Methods

We conducted qualitative interviews with healthcare providers and educators located in clinics throughout the US to assess discrimination and bias that occur in contraceptive care. We recruited potential participants through emails circulated after Continuing Medical Education (CME)-accredited contraceptive trainings provided through the University of California, San Francisco. Training attendees were predominantly women (94%), with race and ethnicity reported as White (54%), Hispanic/Latinx (15%), or Black (14%).^35^ We purposively sampled participants to represent a wide range of healthcare team roles. From 1 August 2022 to 1 July 2024, we enrolled 41 care providers working in contraceptive care, including 11 physicians, 22 advanced practice clinicians (including nurse practitioners, nurse midwives and physician assistants), 5 nurses and 3 counsellors (including social workers and health educators). For those who completed a demographic survey (*N* = 25, 61%), all providers reported their gender as “women” through an open-ended question on the pre-interview survey. Among those that reported their race, participants identified as White (61%), mixed race (12%), Asian (5%) and Latinx (5%; Table 1).

**Table 1.** Key demographic characteristics of providers (*n* = 41)

Characteristic	*N*	%
**Provider type**	** **	** **
Physician	11	27
Advanced practice clinician	22	54
Nurse practitioner	18	44
Physician assistant	3	7
Nurse midwife	1	2
Nurse	5	12
Counsellors	3	7
**Race**		
White, non-Hispanic	25	61
Mixed race	5	12
Hispanic/Latinx	2	5
Asian, non-Hispanic	2	5
Middle Eastern, non-Hispanic	1	2
Unknown race	6	15
**Gender**		
Women	25	61
Gender not reported	16	39

After receiving verbal consent, we conducted 45-minute recorded interviews on Zoom. There were three interviewers (AA, ES, KAD) who took field notes to reflect on their positionality as well as the themes that arose in conversation. We used a semi-structured interview guide covering emerging topics in contraceptive care, including telehealth, discrimination in care, patient counselling scenarios, care for LGBTQ + patients, and experiences since the Supreme Court ruling that removed federal protections for abortion care in the US. Participants received renumeration of a $250 gift card for their time.

### Data analysis

To fill the gap in the lived experiences of gender diversity, we sought the expertise of transgender community members to guide our work.^36^ We developed relationships with experts from the field who informed our interview guide content and approaches to analysis. We invited two community members (AL, FZ), both transgender men, to provide their expertise on anti-transgender biases in contraceptive care as well as their data interpretations. We provided compensation for their time. For contextualising the implications of our interpretations, we also included three healthcare providers (JH, MH, DM) who identify as TGD people living in the US as study authors in the analysis, interpretation, writing and review.

We developed a codebook based on deductive themes of topic areas included in the interview guide, with additional inductive codes that were expanded to capture participants’ responses. First, the team coded two interviews together (AA, CF, ES, KAD, YZ), and then we reconciled differences in coding methodology to streamline methods for applying codes going forward. All coding was completed in Atlas.ti Version 9 (Berlin, Germany). The authors who conducted the interviews (AA, ES, KAD) and the authors who double-coded these data (CF, YZ) are all cisgender women of Latina (AA), Jewish (ES), Middle Eastern (YZ) and White (CF, KAD) descent living in the US.

We analysed the data with full participation of community members (AL, FZ), meeting in regular sessions throughout the analysis and interpretation phases, to explore potential solutions to anti-transgender bias and gender-affirming care that may have been expressed in the participants’ experiences and responses. Utilising a deductive thematic analysis to assess providers’ forms of bias towards providing care for TGD patients, we (AL, CF, FZ, YZ) coded all data together and assigned themes to excerpts focused on implicit biases and preparedness to provide LGBTQ + care. The analysis team identifies as transgender men (AL, FZ) and cisgender women (CF, YZ) living in the US. Differences in coding were reconciled through an iterative approach to ensure full agreement, and the team engaged in analytic discussions to reach a consensus on applications of the selected framework, language used to describe themes and the themes derived.

### Bias framework

**Figure 1.**
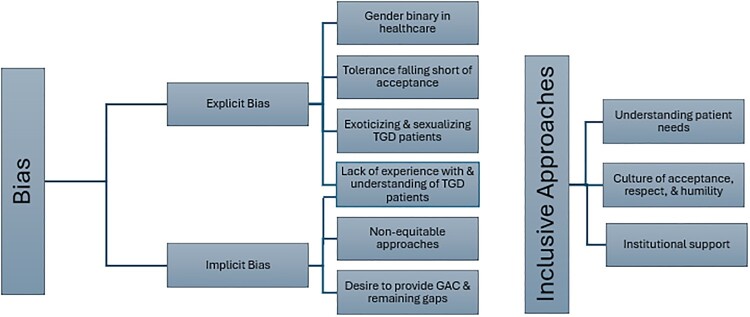
Themes derived from implicit bias, explicit bias and inclusive approaches to care

We utilised a framework of implicit and explicit bias to categorise the ways that bias manifested in contraceptive care.^7^ Explicit bias was operationalised as expressions of exclusion, discrimination and othering of TGD patients described on the part of the provider, the clinic staff and/or the healthcare system. While there are significant overlaps between the manifestations of explicit and implicit bias, the implicit biases here denote the unawareness of cisgender providers in their expressions of subtle forms of “othering” TGD patients. Implicit biases encompassed the more subtle ways that biases affect care, including failing to build rapport with TGD patients and being evident in shifts in providers’ non-verbal communication, such as language and demeanour, when referring to or working with TGD patients. Our community partners selected the language to identify the subthemes within the themes of explicit bias, implicit bias and recommendations found in our data (Figure 1).

The Institutional Review Board at the University of California, San Francisco approved the study (#12-10336 on 30 June 2022) and all participants provided informed consent.

## Results

### Explicit bias

Explicit biases, reflective of pervasive societal norms, are found throughout levels of the healthcare system and result in care that stigmatises and marginalises TGD patients and siloes their care from other providers. Providers in this study named explicit biases that were present through structurally reinforced systems of binarism in health care and large gaps in training. In addition, the identified deficits in providers’ medical knowledge contributed to a workforce that may tolerate, rather than authentically affirm, TGD patients throughout their medical encounters.

#### Gender binary in health care

Providers noted that the clinics and healthcare systems in which they worked were biased against TGD patients. Cis-heteronormativity and gender binarism were prevalent in both electronic healthcare systems and patient intake forms.

One provider also noted that student health centres are further limited by the bureaucracies of registrar and financial aid offices: “*We have some super-outdated stuff on some of the forms we still use. So, we have a lot of that discriminatory stuff … built into our system still*”. The nurse practitioner noted that remedying this process was “*a trickle down of issues*” and “*a beast to try to address*”, where correcting patient pronouns in the electronic healthcare system would then “*go to the registrar and impact their financial aid*”. This hampered the ability of providers in these settings to offer inclusive and affirming care to TGD patients, and they went on to state that: “*We still fall prey to a lot of these very common [things] like messing up people’s pronouns. Messing up their name*” (Nurse Practitioner, #1). Their protocols and forms cause transgender patients to be approached with incorrect names and pronouns, creating a hostile environment for TGD patients.

Providers also discussed structural factors, such as how clinics were physically set up to reflect a gender binary. One nurse practitioner expressed discomfort in caring for TGD patients in settings where their clinic rooms were reflective of the gender binary and separated into “*what they call a male and female exam room*”*.* This provider then described the cis-normative posters present in these rooms: *“They have large graphic images of reproductive organs for visualization and for explanation. And in that situation, I don’t love that. I would love very gender neutral rooms*” (Nurse Practitioner, #2).

#### Limited understanding of, and experience with, TGD patients

Many participants expressed explicit biases that reflected anti-transgender discrimination and stigma, and demonstrated little experience in providing care for TGD patients. Providers had varying degrees of awareness of their own biases and expressed discomfort in their experiences with TGD patients. One physician assistant uncomfortably stumbled through the appropriate terms to refer to a TGD patient: “*But as far as putting a … * *female … * *I guess transgender … * *female, putting a female who is now male on contraception, I’ve never had that before*” (Physician Assistant, #1).

In another example of limited understanding of TGD patients, a nurse practitioner expressed profound confusion by conflating gender and sexuality of their TGD patient: “*It was hard for me to wrap my brain around someone who was born male but they identify more as female, but they’re also interested in females*”. They expressed a micro-aggression that was cis-normative and denoted deviation from sexual normalcy: “*It’s literally a bit of cluster trying to wrap my brain around, are you considered a lesbian female? Is that how you consider yourself?*” This nurse practitioner went on to express a desire in “*trying to learn without being offensive*” (Nurse Practitioner, #3), which serves evidence of the need for provider training on how to provide inclusive and affirming care for TGD patients.

#### Tolerance falling short of acceptance

Several providers expressed a form of tolerance for TGD patients that fell short of full acceptance and advocacy, reflecting deficits in medical knowledge when providing care to TGD patients.

One nurse practitioner noted that their clinic’s patient population was not diverse with respect to gender, despite not collecting that information: “*We don’t see a lot of patients that have diverse gender identities here in the clinic … and I think because we’re not used to asking that*”*.* When patients did disclose their TGD identity, this provider was ill-prepared and surprised: “*it’s something that when it comes up, I always have to check myself with*”*.* They went on to posit that the lack of a gender diverse patient population “*is probably speaking to the discrimination, maybe that’s a barrier. Maybe there’s some reason why they’re not coming into our clinic*” (Nurse Practitioner, #4).

Several providers also noted that a lack of training for the entire healthcare team, from the front desk through medical assistants, created an environment where TGD patients were not fully supported and welcomed. One nurse practitioner described the hostile environment that was created when clinic staff did not use inclusive language with a transmasculine patient: “*I was like oh wow, and I bet this person does not feel welcome coming to this women’s health clinic. And there was some issues at check-in at that appointment and stuff and some confusion with the front desk staff*”*.* They went on to describe how training for inclusive and affirming care should be required for “*the whole staff, even the secretary and front desk needs to be aware of language*” (Nurse Practitioner, #5).

#### Exoticising and sexualising TGD patients

One of the providers that was interviewed explicitly exoticised and sexualised a transmasculine patient. They expressed fetishisation, or a form of acceptability for this patient simply because they were attractive: “*And honestly he probably got the best care because we were so in awe … * *I mean I live in the deep south, we just don’t see that a lot*”. The societal expectations of gender expression are projected onto the TGD patient’s gender presentation and reinforce patriarchal archetypes of gender binarism. This provider’s social acceptance of their TGD patient was grounded in appreciation for the patient’s ability to conform to binary expectations of appearance standards for “males” and “females”: *“And we’re in awe of like I mean he’s a good-looking man. I was like what, like this is crazy. So he probably received actually a lot of positive attention … *” (Nurse Practitioner, #6). While this provider was an outlier in our dataset, we note that this is common in the experiences of TGD patients.

### Implicit bias

Implicit biases reinforce a cis-normative understanding and delivery of providers’ clinical care. Providers showed a lack of awareness of the unique individual needs of TGD patients, although a subset recognised their own limitations and lack of skills and approaches to provide gender-affirming care.

#### Lack of understanding of, and experience with, TGD patients

Several providers shared a perception that they did not care for many transgender patients. These and other participants relayed experiences that revealed both implicit bias and a limited understanding of gender diversity. These forms of implicit bias contribute to cisgender providers' “othering” TGD patients through a lack of awareness of their own cis-normative behaviours and language. One nurse practitioner’s response to our inquiry about training needs included othering language by referring to transgender patients as “those”: “*I will say mostly taking care of transgender patients, because we don’t have a lot of those*” (Nurse Practitioner, #7).

Providers also frequently misused language to refer to TGD patients and/or lacked the correct terminology to refer to gender-affirming surgeries. Some of the language providers used elucidates a lack of medical knowledge around patient preferences for transitioning by referring to gender-affirming surgeries as “*switch their genetic, genital site*” (Nurse Practitioner, #8). Additionally, the conflation of gender identity with sexuality was common in our study sample and revealed shortcomings in providers’ understanding of the differences between sex, gender, gender expression and sexuality. The most prominent form of implicit bias surrounding TGD patients was the misuse of pronouns for patients. There were several instances when providers failed to use identity-congruent pronouns; for example, though affirming language would have included the use of “he/him” or “they/them” pronouns when referring to transmen accessing contraceptive care, many providers continued to use incorrect “she/her” pronouns to refer to these patients. One nurse practitioner’s reflection about a patient “*who was born female and wanted to go through, um, the transgender change*,” included multiple instances of misgendering: “*She was letting us know that her parents were on board with it, and we gave her a referral to a clinic that would be able to provide her hormone treatments to help her*” (Nurse Practitioner, #9).

#### Non-equitable approaches

Some participants' expressions of inclusiveness reflected a “one-size-fits-all” approach to providing contraceptive care for TGD patients. Providers may see themselves as approaching TGD patients neutrally in that they “*don’t even think about their choices of sexual behavior or choices to provide care for them*” (Nurse Practitioner, #8) or that “*in terms of counseling, I don’t know that I would approach it any differently though. Just because of their status or what they … * *are*” (Nurse Practitioner, #10). However, these examples illustrate their failure to treat a TGD patient as an individual with unique circumstances, needs, preferences and goals. One nurse practitioner further emphasised their neutrality by expressing, “*They’re human, I am going to take care of them just like I would expect someone to take care of my mom, my dad, my siblings*” (Nurse Practitioner, #8). This exemplifies the absence of effort to tailor care to each patient, a cornerstone of patient-centred care, and to recognise the gender-specific needs of TGD patients.

These non-equitable approaches may reduce the ability to perceive and respond to the individual and gender-specific needs of TGD patients. In another example, a nurse practitioner expressed that delivering contraceptive care requires specific training: “*just the knowledge of what each contraception does, because if you don’t know what they do, it’s hard to prescribe because you don’t know anything about the medication itself*”. This provider then demonstrated a lack of cultural competency and sensitivity by expressing that TGD patients “*just have different lifestyle choices*”*.* They also failed to contextualise how contraceptive counselling and care may differ for TGD patients and diminished the diversity of perspectives and contraceptive needs of the unique individuals within TGD populations: “*regardless of how the patient identifies, if their body structure is female, I would treat that like I would do any other female*” (Nurse Practitioner, #7).

#### Desire to provide gender-affirming care and remaining gaps

While several providers expressed supportive attitudes and demonstrated allyship for patients that identified as TGD, providers’ statements simultaneously demonstrated large gaps in medical knowledge and cultural humility. Many of these gaps signified a lack of training, skills and approaches for providing contraceptive care. One provider’s comment highlighted an important question regarding patients receiving gender-affirming hormone treatments, such as testosterone: “*If they’re taking certain hormones, is giving a contraceptive hormone going to be something that may be harmful to them?*” (Physician Assistant, #1)*.* Another provider expressed hesitation in providing contraceptive care related to their own gaps in knowledge about medical contraindications for patients using testosterone: “*So she wants something to prevent her from getting pregnant, yet she may be also on testosterone or something, just being able to know how they would interact or how would that cause a potential health risk for increase for blood clots and things like that, which is patient safety*” (Nurse Practitioner, #7).

In addition, providers expressed a preference to refer patients out to receive gender-affirming care rather than to integrate it into their practice. Providers also shared assumptions that patients were receiving gender-affirming care elsewhere, which may create gaps in care for patients seeking contraceptive care: “*I would hope that the endocrinologist taking care of all that is going to bring up contraceptive care. And maybe that’s why we don’t do that, because they’re being taken care of outside of here*” (Physician Assistant, #1).

Several providers expressed the desire to have the language for contraceptive counselling for gender diverse patients, but felt they lacked the tools to provide this care. One registered nurse described the anxiety around providing care for TGD patients:

*“There is like * … * a type of anxiety or like anxiety of the unknown when we do have patients that identify as transgender or queer or * … * um * … * we do have * … * like our staff * … * doesn’t know what word to use * … * when talking to patients. The idea of asking people for their pronouns is still pretty uncomfortable for some of our staff. But there’s been ongoing trainings that our clinic has put into place to help us understand more how to address patients and care for their needs in that way” (Registered Nurse, #1).*

### Inclusive care for TGD patients

Some participants had observed implicit and explicit biases within their workplaces and shifted towards advocacy for trans-inclusive and trans-affirming solutions both on individual and institutional levels. Participants also described how understanding patients’ needs allows them to support a culture of care and support for TGD patients.

#### Understanding patient needs

In delivering inclusive care, providers expressed ways to build rapport with TGD patients through gender-affirming sexual history taking and gender-affirming care. These approaches demonstrated an understanding of medical needs without conflating gender with body parts or making assumptions about the anatomy of patients and their partners or a TG/GE person’s identity, practices or desires around contraception or sexual health*.* One physician exemplified cultural sensitivity by stating “*There are patients who present one way, and maybe more masculine, doesn’t mean that they have a partner who … it doesn’t in any way indicate what kind of partner they have and what activities they could be doing and is there pregnancy possibilities in their actions*”*.* They went on to express that using gender-affirming language in delivering inclusive care is achievable: “*You’re just asking like very neutral questions. It’s only weird if you make it weird*” (Physician, #2).

#### Culture of acceptance, respect and humility

Some providers exemplified a culture of inclusivity that treats TGD patients with acceptance, respect and humility. Providers reported actively seeking ways to build trust with patients and offer full acceptance of a patient’s gender. One registered nurse expressed how building rapport and trust with TGD patients takes time:

*“I think it really comes down to * … * making them feel comfortable … . … I want to make sure that they feel like they can share those pieces of their identity or like those pieces of their health management … . And I think creating that trust was a really difficult thing and we often ask our patients to be trusting in us before we’ve actually done the effort to building trust” (Registered Nurse, #2).*

The intentionality of their person-centred contraceptive care delivery illustrates explicit indicators of inclusion and safety. One nurse explained how their intention has the power to interrupt patients’ initial expectations of receiving sub-par care: *“Because you can be the nicest person ever and use the right pronouns and be very kind and loving, but then there’s also that other level of showing them respect by educating yourself on what their needs might be”* (Nurse Practitioner, #11).

Providers also reported that TGD patients can feel welcomed when providers recognise patients as the experts in their own experiences. One provider also noted the experiences of gender joy that patients may experience when receiving gender-affirming contraceptive care:

*“I’ve seen the relief on patient’s faces when they know they’re accepted maybe for the first time, when they know we’re going to use their correct pronouns. And if they know if we are not judging or making them uncomfortable or anything like that, we’re asking about their lives and their partners. It’s just a normal conversation and you can just feel the sense of relief and comfort” (Nurse Practitioner, #12).*

#### Institutional support

Providers reported ways that institutional advocacy supported their individual efforts to provide gender-affirming care. For example, in creating a more inclusive contraceptive care environment, some mentioned removing the gendered wording of clinics’ names, rooms and procedures and instead: “*added ‘people’ or ‘individuals’ or instead of ‘well woman exams’, ‘wellness exams*’”. This social worker went on to describe how they turned to a non-binary co-worker to further deliberate institutional gaps: “*we will discuss or sometimes roll eyes or that kind of thing about some of our materials and how they feel like they haven’t really caught up to us*” (Social Worker, #1).

Further, some described how removing burdens in navigating paperwork that is gendered or incongruent with person-centred care creates more inviting healthcare spaces for TGD patients and explicitly communicates welcoming and belonging. Another social worker went on to describe outdated clinic materials: “*some of our paperwork has like, ‘Does the man involved in your decision know about this?’ And we have plenty of people that are nonbinary or there’s not a man involved in their decision*” and how they advocated for more approachable language from their institution: “*So, I am telling the people above me to change that paperwork*” (Social Worker, #2).

## Discussion

In this paper, we analysed explicit and implicit forms of anti-transgender bias among participants from a wide range of providers working in contraceptive care in the US. We uncovered the ways that contraceptive care providers uphold, express, and internalise anti-transgender biases that are prevalent in both the healthcare system and broader society. Explicit biases included a lack of understanding and experience with TGD patients’ gender identity, pronouns, and medical needs. This contributed to a culture of tolerance and non-acceptance within a non-inclusive atmosphere for TGD patients. Explicit bias was further exemplified by the pervasiveness of gender binarism within the healthcare system. Implicit biases included large deficits in both medical knowledge and culturally humble approaches to care, as well as “equal” but *inequitable* approaches to providing person-centred care for TGD patients as compared to cisgender women. We also uncovered strategies to mitigate these biases, as providers in this sample also discussed several recommendations to address the presence of anti-transgender bias in contraceptive care. In some instances, intentionality in understanding TGD patients’ needs helped providers shift into a culture of acceptance, respect, and humility when working with TGD patients. Providers also emphasised the need for multi-level solutions, such as institutional and system-level applications of gender-neutrality, which help make clinics more trans-inclusive and trans-affirming. Given the historical and present-day sociopolitical hostility towards TGD patients, contraceptive care spaces are a critical avenue for affirming and welcoming TGD patients in the healthcare system and for upholding these patients’ right to access quality, person-centred sexual and reproductive health care.

Providers’ lack of medical knowledge perpetuates disparities among TGD patients in reproductive health care.^18^ In the absence of providers and clinic staff demonstrating social safety cues, many TGD patients will continue to experience contraceptive care settings as exclusionary and unsafe. Our findings are consistent with a large body of research demonstrating that TGD patients frequently encounter confusion, discomfort and distrust from healthcare providers.^5,19,20,29,30^ TGD patients are often unfairly burdened with educating their providers.^37^ Further, deficits in providers’ medical knowledge perpetuate harms and are a strong predictor of health care non-utilisation for TGD patients.^22,38^ Previous studies have found that 6–9% of providers incorrectly counsel TGD patients that testosterone therapy is contraception, which may lead to an undesired pregnancy.^27,39^ Providers may also restrict access to the full range of contraceptive options available to TGD patients, stemming from the misconception of interactions with gender-affirming hormones.^22^ There were several instances in which contraceptive care providers suggested that other specialists were better equipped at providing gender-affirming care. These referrals or denials of care at the very least create delays in care and gaps in the healthcare system, where TGD patients may not receive timely care or get care at all.^19,20^

Persistent implicit provider bias undermines providers’ efforts to operationalise person-centred care and ultimately contributes to lower quality of care and perpetuates health disparities.^40,41^ Providers in this sample also exhibited bias by misgendering patients and often projecting cis- and hetero-normative principles onto TGD patients’ gender and sexual identities.^42^ Gender-affirming sexual history taking requires an understanding of medical needs with respect to sexual and reproductive health without conflating gender with body parts or sexuality. In the absence of knowledge of the anatomy of patients and their partners, not making assumptions about a TG/GE person’s identity, practices or desires around contraception or sexual health is an essential first step in understanding each patient’s unique medical needs.^25^ However, many providers in this study failed to treat TGD patients as individuals with unique circumstances, needs, preferences and goals, thereby missing the opportunity to provide care that is tailored to each patient, a cornerstone of patient-centred care. Further, trans-inclusive care supports the authentic use of patients’ correct names and pronouns, a respect for a variety of relationship styles and choices, inclusive record keeping, and a fluency of terminology and understanding of gender and gender identity as distinct from issues of sexual behaviour and sexual identity.^25,30,43^ Gender-affirming care is within the scope of contraceptive care providers, and the inclination and desire to exhibit medical knowledge and allyship with TGD patients denotes an important need in creating safe learning spaces for providers to explore questions.^44^

It is paramount to recognise that providers operate within institutions, and that institution-level factors play a significant role in hindering or facilitating the delivery of equitable care to TGD patients.^3^ Participants offered a few examples of workplace culture that emphasise inclusion, through the gender-inclusive influences of paperwork, names of both clinics and procedures and exam room conventions. These providers identified how structural influences impact provider decision-making, making it easier for them to offer gender-affirming care within a congruent environment that reflects intentional inclusion, safety and respect – in contrast to the predominate system of “two-gender” or binary medicine, which is rife with systemic failures to serve TGD patients.^45^ TGD patients have unique needs in accessing health care, and, alongside principles of trauma-informed care, accommodations for disclosure, examination, procedures and treatment must be offered. Creating a trans-inclusive clinical atmosphere requires structural and systemic investments in waiting room environments and restroom access, in addition to staff training. For example, removing the language of “women’s health” from gynaecological care effectively reduces the need for TGD patients to access and navigate reproductive health care exclusively through spaces that are gendered.^25,30^ Without institutional supports and/or organisational changes, individual provider efforts to be inclusive of TGD patients may fall flat. Institutional commitment to training must set these expectations for all staff.^3^ In order to create a gender-affirming environment, ongoing and mandatory training for all staff, at all points throughout a patient encounter, is needed.^25,31,46^ Incorporating the pedagogies of philosophy and sociology in training may further support providers in unpacking the concept of gender and binarism outside of the medical system to expand their learning and understanding of gender diversity.^47,48^ However, training alone does not improve the inequitable care and stigmatising experiences of TGD patients in the healthcare system.^49^

There are important limitations to this work. We have limited data on providers’ sexual and gender identities (61%). Among those who reported their gender identities, all reported their gender as “woman”, but we did not elicit more information regarding gender, such as cis, trans or other.^30^ We are committed to improving our data collection methods in the future to align with current recommendations on eliciting gender identity.^50^ In addition, we recruited providers who attended contraceptive trainings and those that participate in educational activities, as well as in research, may be inherently different from other providers in the field. We also did not include in our analysis the potential differences by provider type. Lastly, we did not include community members in the conceptualisation of this project and are therefore lacking the full breadth of community-based and -led research. Our community members were heavily involved in both thematic analysis and deriving themes, as they have lived experience in their communities.

## Conclusions

Biases specific to TGD individuals are perpetuated through a lack of staff and provider awareness and experiences of stigmatisation in contraceptive care settings. Our data elucidate large gaps in medical knowledge and the importance of staff training to facilitate understanding of the diverse experiences of TGD persons. Performative social inclusion and training alone fall short of addressing the injustices and inequities that TGD patients experience in the healthcare system. Our findings highlight multi-level areas to focus advocacy to increase awareness of bias, dispel confusion on how to provide high-quality gender-inclusive contraceptive care with updated research and evidence, and to integrate structural changes within clinics to reduce the burdensome impacts of bias on TGD patients. Cisnormativity among providers as well as in clinic policies and practices must be addressed to promote trans-inclusive contraceptive care and reduce critical barriers to person-centred care for individuals of all genders.

## Author contributions

Methodology: YZ. Project management: YZ. Formal analysis: YZ, CF, AL, FZ, AA, ES, KAD, MH, DM, JH. Investigation: YZ, CF, AL, FZ, Data curation: AA. Funding acquisition: CH. Conceptualisation: CH. Supervision: CH. Writing – original draft: YZ, CF Writing – review & editing: YZ, CF, AL, FZ, AA, ES, KAD, MH, DM, JH, CH.
